# Sanjad-Sakati Syndrome Dental Management: A Case Report

**DOI:** 10.1155/2013/184084

**Published:** 2013-02-21

**Authors:** Hisham Y. El Batawi

**Affiliations:** Pediatric Dentistry, Sharjah University City, P.O. Box 27272, Sharjah, UAE

## Abstract

Sanjad-Sakati syndrome (SSS) is a rare genetic disorder with autosomal recessive pattern of inheritance characterized by hypoparathyroidism, sever growth failure, mental retardation, susceptibility to chest infection, and dentofacial anomalies. A child with SSS was referred to the dental departmentseeking dental help for sever dental caries which was attributed to his dietary habits and quality of dental tissues. Full restorative rehabilitation was done under general anesthesia. Two years later, the child presented with recurrent caries affecting uncrowned teeth. High carries recurrence rate was blamed for the nutritional habits endorsed by the parents. Only steel crowned teeth survived such hostile oral environment which suggested shifting of treatment strategy towards full coverage restorations instead of classical cavity preparations and fillings during a second attempt for dental treatment under general anesthesia and for the dental treatment of two cousins of the same child. The author recommends effective health education for parents including the nature of their child's genetic disorder, nutritional needs, and dental health education to improve the life style of such children.

## 1. Introduction

 Sanjad-Sakati syndrome (SSS) is a rare autosomal recessive disorder (OMIM 241410) that is confined to Arab Middle Eastern populations. It is characterized by congenital hypoparathyroidism, hypocalcemia, seizures, hyperphosphatemia, growth retardation, dwarfism, mental retardation, and dysmorphic craniofacial features including microcephaly, deep-seated eyes, depressed nasal bridge, and micrognathia [1].

### 1.1. Previous Reports

#### 1.1.1. Oman

Al-Ghazali and Dawodu [2], in 1997, reported a case in Oman with the a forementioned features. Computerized tomography (CT) rain scan showed immature myelination suggesting that failure of growth is due to hypothalamic origin. 

Thirteen years later, Rafique and Al-Yaarubi [3] claimed the first report of SSS in Omani children where they reported SSS in three siblings (two girls and one boy). The authors highlighted the need for routine DNA counseling for early diagnosis and prevention of associated comorbidities. 

#### 1.1.2. Palestine

In 2006, AbuDraz [4] reported two unrelated Palestinian children; both of them had the syndrome's manifestations plus small sized atrial septal defect detected by echocardiogram.

#### 1.1.3. Israel

Platis et al. [5], in 2006, reported one 12-year-old child of Bedouin origin with the syndrome. The child was a product of consanguineous marriage. 

#### 1.1.4. Qatar

In 1990, Richardson and Kirk [6] reported eight Qatari children, four boys and four girls, all born to consanguineous parents. Seven of these children had medullary stenosis of long bones. 

#### 1.1.5. Saudi Arabia

Sanjad et al., in 1991, reported 12 infants to have the syndrome; eleven of these infants were the product of consanguineous marriages while four has similarly affected siblings. 

 In this paper, we describe the concerns encountered in dental management of a child with the syndrome and the modifications in treatment plan that had to be done in managing two of his cousins with the same disorder. 

## 2. Case Report

A four-year-four-month-old boy belonging to a known tribe in Western Province of Saudi Arabia was referred to the dental department of a private hospital in Jeddah, Saudi Arabia. The child's pediatrician diagnosed the case as Sanjad-Sakati syndrome whose elder brother had the same condition and died at the age of 12 years from severe pneumonia. The child's medical history revealed that he was born at the same hospital with birth weight 1729 grams, occipital-frontal circumference 29 cm, serum calcium 1.5 mm/L, and phosphate 2.6 mmol/L. Walking and speech were delayed as reported in the medical record. 

### 2.1. Pediatric Management

The child had frequent hospitalizations due to recurrent pneumonias. Oral calcium supplements, vitamin D, and Polycose supplement (Abbott Nutrition) were administered in addition to symptomatic anticonvulsive drugs. No growth hormone therapy was administered by pediatrician as it showed no effect on the late older brother. 

**Figure 1:**
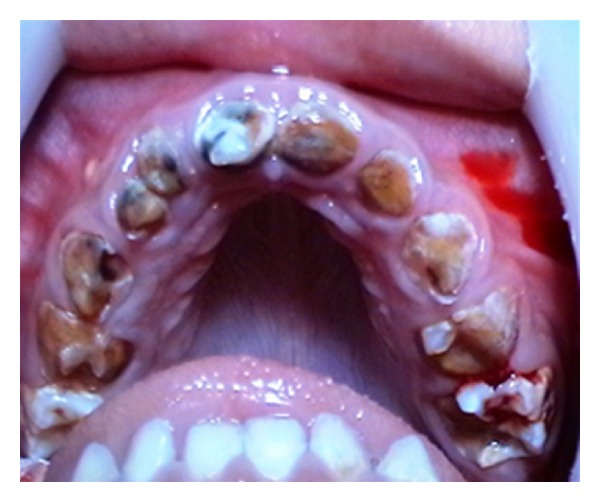
Preoperative Sanjad-Sakati intraoral features.

The child showed typical facial features of short stature, deep sunk eyes, micrognathia, depressed nasal bridge, relatively large ears, and mental retardation.  Figure 1 shows an 11-year-old cousin girl of the child with SSS. 

### 2.2. Intraoral Findings

**Figure 2:**
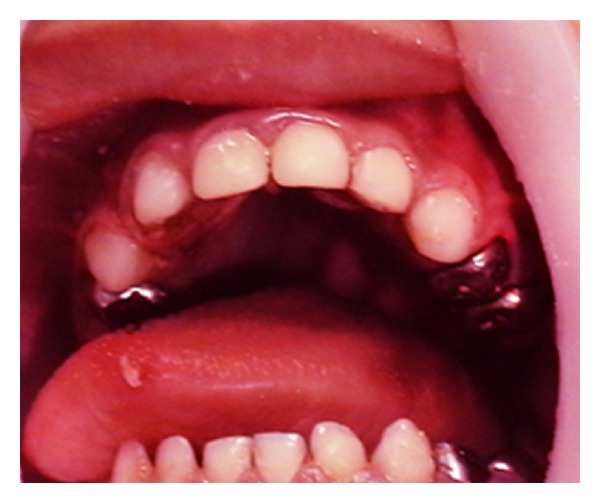
Postoperative restorative first attempt.

The child presented with sever dental caries and neglected oral hygiene, all upper teeth were badly decayed, and lower molars were also exposed while lower anterior teeth were minimally affected which suggests a nursing bottle pattern of dental caries. High vaulted palate, micrognathia, dental anomaly of extra upper right lateral deciduous incisor, and microdontia were observed in first deciduous molars as well (Figure 2). 

### 2.3. Dental Treatment Plan

Full dental treatment was planned including pulpotomies, steel crowns, restorations, extraction of supernumerary upper right lateral incisor, and parents education regarding nutrition and oral hygiene. Considering the amount of work needed versus the patient's cooperation limits, a decision was made to perform the procedure under general anesthesia.

### 2.4. Patient Privacy

 Informed consents were obtained conforming to the Joint Commission for International Accreditation (JCIA) standards and following the World Medical Association Declaration of Helsinki on Ethical Principles for Medical Research Involving Human Subjects, October 2001. 

In a conservative social environment like Saudi Arabia, the author had to get a special consent regarding publication of the three pediatric cases involved in this paper. All parents of the three children permitted publication while only one parent permitted one photograph for publication.

### 2.5. Anesthesia Management

Preoperative antibiotic prophylaxis was administered for pneumonia concerns. The patient's cooperation level did not allow for intravenous line insertion; accordingly, induction was carried out via facemask with 3% Sevoflurane, Abbott Co., in 100% oxygen. Following insertion of intravenous line, Propofol, Diprivan, AstraZeneca Co., was administered. Nasotracheal intubation was done smoothly with aid of external laryngeal manipulation. During the course of dental procedure, Dexamethasone was given to aid in reducing oral edema. The procedures were done in 130 minutes excluding induction and extubation. During dental management, oxygen saturation was 98-99% and heart rate was 90–125 with the highest reading during dental pulp tissue extirpation. Postoperative recovery was uneventful, and the child was discharged the next day after, pediatric verification that he is fit for discharge.

## 3. Discussion

Al-Malik, in 2004 [7], reported the oral findings associated with SSS, which included micrognathic mandible and maxilla thin lips, high arched palate, severe dental caries, microdontia, and enamel hypoplasia. In the reported case, all intraoral symptoms reported by Al-Malik were observed with exception of enamel hypoplasia. The parents gave history that the teeth erupted with whitish chalky appearance which turned soon into big cavities and chipping. That statement might be more attributed to the rampant caries resulted from nursing bottle use or early childhood caries than to enamel hypoplasia. 

The autosomal recessive pattern of inheritance of hypoparathyroidism resembles that observed in Kenny-Caffey syndrome which is manifested as growth retardation, craniofacial anomalies, small hands and feet, hypocalcaemia, and hypoparathyroidism in addition to radiographic evidence of cortical thickening in long bones; the latter is not reported in SSS. Both syndromes are thought to be allelic as they have been mapped to the same chromosome, share an ancestral haplotype, and both are chaperon diseases caused by a genetic defect in the tubulin [8]. 

In Arab populations, the deep rooted norm of consanguineous marriage has been widely blamed as a predisposing factor for autosomal recessive diseases such as SSS [9]. The reported case is a product of consanguineous marriage, and two years later, two of the cousins of that child patient were referred to undergo full dental rehabilitation under general anesthesia. 

### 3.1. General Anesthesia

Wasersprung et al. [10], in 2010, reported dental treatment of an SSS child under general anesthesia that went uneventful except for episodes of desaturation (80% SaO_2_) that was managed by bronchodilators and ventilation. Al-Malik, in 2004, reported uneventful anesthesia management for an SSS case. In our paper, the child was anesthetized twice with the second time after two years. Both instances went uneventful with no need for intensive care stay. Prophylactic antibiotic coverage was administered to help avoid the risk of chest infection. Tube selection was decided according to the child's weight rather than the child's age. 

### 3.2. Dental Management

**Figure 3:**
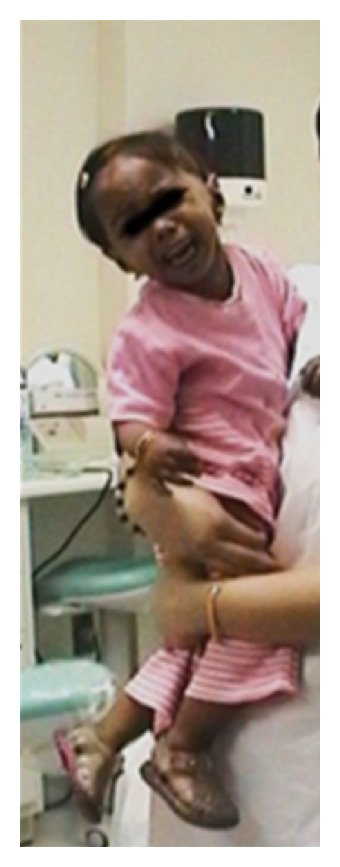
A Sanjad-Sakati 11-year-old girl.

Our patient has been subjected to dental treatment under general anesthesia two times with an interval of two years. The aim of the first dental treatment was to restore back all decayed teeth to a nearly normal condition in addition to preventive dental treatment including sealants and fluorides as well. The dental procedures were considered as part of total dental care that included dental health education to the parents regarding nutritional and oral hygiene habits (Figure 3). 

After two years, the child appeared with severe recurrent caries in all composite resin filled teeth, with periapical infection related to the upper four incisors. Radiographic examination was not possible due to the patient's limited cooperation and the possible trauma involved during placing the X-ray sensor in such a small oral cavity. All steel crowned teeth were in satisfactory clinical state after the two-year postoperative period.

 The parents stated that they failed to quit night bottle feeding and that the child has no muscular strength to chew rough fibrous food. Accordingly, a change of strategy was decided. A second dental rehabilitation under general anesthesia was decided. The second dental management stressed on steel crowns and dental extractions more than restorative procedures, that is, shifting toprocedures with more solid prognosis to minimize the child's future dental needs. Further, dental health education concentrated on strict dental hygiene, avoiding night feeding and shifting to healthier semisolid food choices. Strict followup schedule was attempted on monthly bases where dental prophylaxis was performed in each monthly visit. 

The same strategy was followed with two cousins of the same child and proved satisfactory on monthly follow-up bases for two years. Outcome assessment was based on parental satisfaction questionnaire and lack of pain in addition to periodic clinical evaluation. Postoperative body weight monitoring was carried out. No significant postoperative increase in body weight could be observed.

Dental management for SSS children was reported by Al-Malik in 2004 and Waserprung et al. in 2010. No comparisons of fillings versus crowns regarding their longevity were mentioned in both reports. As Waserprung et al. reported anodontia of 12 permanent teeth upon radiographic examination, in our case, X-ray examination was not possible. This does not exclude the possible presence of anodontia in our case which in turn supports our recommendation for the use of steel crowns instead of fillings to prolong the service life of decayed teeth in SSS children. 

The use of preformed steel crowns to restore teeth with microdontia has encountered some difficulties especially in lower first primary molar. In our case, we used upper opposite side crowns to restore lower first primary molars due to the smaller mesiodistal width of upper deciduous molars than their corresponding lowers. 

Due to the conservative nature of Middle Eastern people in general and Saudi population in particular, the three figures included in this paper were the only photographs approved by the parents for publication. The same cultural habits and traditions may have negative effect on parents preventing genetic counseling which raises concerns regarding proper documentation and reporting of cases as well as incidence studies.

## 4. Conclusion

The author underlines the importance of premarriage genetic counseling and education related to consanguinity and reproductive health in the Arab world. Constructive teamwork management is a must for successful management of children with Sanjad-Sakati syndrome. 

Children with Sanjad-Sakati syndrome lack the growth and development of proper masticatory apparatus which renders them in continuous need for soft and semisolid food. This constitutes a challenge for their restorative dental treatment. This paper recommends the use of steel crowns if possible. Dental treatment should be directed to modalities with unquestionable prognosis and try to prolong the durability of primary molars to improve mastication and dietary habits, thus promoting the child's general health and quality of life. 

Dental health education for parents of SSS children regarding nutrition and oral hygiene is of prime importance as well as watchful postoperative followup.
